# Sustainability capacity of a vegetable gardening intervention for cancer survivors

**DOI:** 10.1186/s12889-022-13644-5

**Published:** 2022-06-22

**Authors:** Mallory G. Cases, Cindy K. Blair, Peter S. Hendricks, Kerry Smith, Scott Snyder, Wendy Demark-Wahnefried

**Affiliations:** 1 University of California San Francisco Helen Diller Family Comprehensive Cancer Center, San Francisco, CA USA; 2 Department of Internal Medicine, University of New Mexico, MSC07-4025, Albuquerque, NM 87131 USA; 3 University of New Mexico Comprehensive Cancer Center, Albuquerque, NM USA; 4 Department of Health Behavior, School of Public Health, University of Alabama at Birmingham, Birmingham, AL USA; 5 Alabama Cooperative Extension System, Auburn University, Auburn, AL USA; 6 School of Education, University of Alabama at Birmingham (UAB), Birmingham, AL USA; 7 Department of Nutrition Sciences, School of Health Professions, UAB, Birmingham, AL USA; 8 O’Neal Comprehensive Cancer Center at UAB, Birmingham, AL USA

**Keywords:** Sustainability, Program sustainability, Evaluation, Cancer survivor, Neoplasms, Health behavior, Gardening, Cancer

## Abstract

**Background:**

Health behavior interventions, especially those that promote improved diet and physical activity, are increasingly directed toward cancer survivors given their burgeoning numbers and high risk for comorbidity and functional decline. However, for health behavior interventions to achieve maximal public health impact, sustainability at both the individual and organizational levels is crucial. The current study aimed to assess the individual and organizational sustainability of the *Harvest for Health* mentored vegetable gardening intervention among cancer survivors.

**Methods:**

Telephone surveys were conducted among 100 cancer survivors (mean age 63 years; primarily breast cancer) completing one-of-two *Harvest for Health* feasibility trials. Surveys ascertained whether participants continued gardening, and if so, whether they had expanded their gardens. Additionally, surveys were emailed to 23 stakeholders (Cooperative Extension county agents, cancer support group leaders, and healthcare representatives) who were asked to rate the intervention’s ability to generate sustained service and produce benefits over time using the eight-domain Program Sustainability Assessment Tool (PSAT).

**Results:**

The survey among cancer survivors (91.9% response rate) indicated that 85.7% continued gardening throughout the 12 months following intervention completion; 47.3% expanded their gardens beyond the space of the original intervention. Moreover, 5.5% of cancer survivors enrolled in the certification program to become Extension Master Gardeners. The survey among stakeholders generated a similar response rate (i.e., 91.3%) and favorable scores. Of the possible maximum of 7 points on the PSAT, the gardening intervention’s “Overall Capacity for Sustainability” scored 5.7 (81.4% of the maximum score), with subscales for “Funding Stability” scoring the lowest though still favorably (5.0) and “Program Evaluation” scoring the highest (6.3).

**Conclusions:**

Data support the sustainability capacity of the *Harvest for Health* vegetable gardening intervention for cancer survivors. Indeed, few interventions have proven as durable in terms of individual sustainability. Furthermore, *Harvest for Health*’s overall organizational score of 5.7 on the PSAT is considered strong when compared to a previous review of over 250 programs, where the mean overall organizational PSAT score was 4.84. Thus, solutions for long-term funding are currently being explored to support this strong, holistic program that is directed toward this vulnerable and growing population.

**Trial registration:**

ClinicalTrials.gov Identifier: NCT02150148

## Background

The steadily increasing number of cancer survivors, who now comprise 5.0% of the U.S. population [1], are faced with greater risk for chronic diseases (e.g., cardiovascular disease, and diabetes) and second malignancies. Many chronic diseases and cancer are associated with a higher likelihood of functional limitations [1–3]. Adoption of healthy lifestyle behaviors has the potential to decrease risk for both disease and functional decline. However, for lifestyle changes to effectively result in tangible health benefits, the effects must be durable on an individual level. Moreover, the intervention or program must have the potential for sustainability in terms of organizational capacity [4, 5]. Recent reviews suggest that few diet and exercise interventions among cancer survivors measure long-term durability, and of those that do, results show relatively modest long-term improvements [6–8]. Furthermore, many of the studies reviewed implement follow-up periods that are fairly brief (e.g., 3 months), and are criticized for poor reporting standards, and a high risk of bias. Additionally, many studies include circumscribed samples that are largely comprised of younger White women with breast cancer [6, 8], and hence ungeneralizable to the larger population of survivors of whom 60% are age 65 and older [1].

A program centered around a home-based, vegetable gardening intervention may prove durable, as it takes place at home, where people may be especially motivated to “nest,” i.e. nurture and cultivate their immediate surroundings. Additionally, people need only step outside their front or back door, thus limiting travel (required only occasionally for supplies, or perhaps not at all if supplies are delivered). Also, since the vast majority of cancer survivors are seeking complementary and alternative medical therapy to improve their health [9], a holistic approach, such as a gardening intervention, is likely to appeal to this population. Furthermore, participants are less likely to lose interest when the intervention ends since gardening has inherent prompts (i.e., watering, weeding, pest control, pruning, harvesting), which may prevent boredom common with other diet and exercise programs [10, 11].

Maintaining programs over time is increasingly difficult even for the most beneficial and successful of interventions. Program benefits can only be delivered if these programs can be sustained. In many settings resources for public health programs are limited and decreasing [12]. Sustainability at the organizational level requires that the health behavior intervention (or program) becomes institutionalized within an organization in non-research settings, such as schools, clinics, or community-based organizations.

The growing field of implementation science has increased the amount and diversity of research in the area of “maintaining programs and their benefits over time,” or sustainability [13, 14]. Program sustainability, as a concept, is not new with theoretical work present in many fields (e.g., business, health care administration, social services, public health) [15]. Once programs have been implemented, however, much less attention is often paid to the program’s overall outcome [16]. However, intended benefits are dependent upon the program reaching full maturity. By better understanding what encourages sustainability and the factors that contribute to it long-term, we can better benefit from the investment in public health research and program development [17].

We evaluated the capacity for sustainability of two *Harvest for Health* vegetable gardening interventions tested for feasibility among cancer survivors. Both interventions were delivered in partnership with the Cooperative Extension Master Gardener Program to facilitate one-on-one mentorship of cancer survivors in planning and maintaining three seasonal vegetable gardens over 12 months as well as providing gardening supplies, plants, and seeds. The intervention aimed to improve diet, physical activity, and physical functioning [10, 18–20]. In undertaking this research, we explored sustainability from both individual and organizational perspectives.

## Methods

### *Harvest for health* vegetable gardening intervention

*Harvest for Health* is a 12-month home-based, mentored vegetable gardening intervention for cancer survivors. Designed with dissemination and sustainability in mind, the intervention capitalizes on an extant infrastructure — the Cooperative Extension System (Extension) — operated by land-grant universities across the U.S. The Master Gardener Program is one of the more popular education and outreach programs offered through the Extension and exists in all U.S. states and territories. In Alabama specifically, there are 44 county and regional Master Gardener Associations with about 1,800 new and veteran volunteers in total.

Through a certification process, the Master Gardeners receive research-based training in horticulture with the expectation that they will use this training to serve their communities. In testing feasibility, *Harvest for Health* was offered as a community service project that Master Gardeners in Alabama could select as part of their volunteer hours.

In *Harvest for Health*, survivors are individually matched with a Master Gardener (based on geographic proximity) who works with them to plan, plant, tend, and harvest three vegetable gardens over the course of a year. Via grant support, survivors were given an estimated $500 of gardening supplies, which included a raised bed garden or four garden boxes, gardening tools and supplies, and plants and seeds. The participants were able to keep all the gardening materials to encourage continued gardening upon completion of the intervention.

The feasibility of *Harvest for Health* was evaluated in two randomized controlled trials employing a waitlist-controlled design. Detailed methods and results have been published [18–20]. A summary of the two feasibility trials is included in Table 1. Both of these trials served as a means to collect individual sustainability data on the gardening intervention. Approval for the original studies, as well as the protocol amendment that enabled further follow-up, was obtained from the University of Alabama at Birmingham Institutional Review Board (X130603001 and X130626006).

**Table 1:** Description of the harvest for health feasibility trials

	Birmingham Breast Cancer Survivors Study	Alabama Senior Cancer Survivors Study
Years conducted	2013 - 2016	2014-2015
Setting	Birmingham, Alabama (metropolitan area)	Alabama (urban and rural counties)
Study design	Randomized controlled trial – waitlist control	Randomized controlled trial – modified waitlist control ^a^
Length of trial	1 year	1 year
Age	No limitation	Age 60 years and older
Cancer type(s)	Female breast cancer survivors	Cancer survivors with a cancer type/stage associated with a 60% or greater 5-year relative survival rate ^b^
Number enrolled in trial / completing trial	82 / 78	46 / 42
Number contacted / completing telephone survey	78 / 71	22 / 20

^a^The 22 older cancer survivors randomized to the waitlist control group did not receive the full, intensive intervention, and thus were not included in the follow-up telephone survey

^b^Cancer types include: in situ bladder; loco-regional staged: breast (female), Hodgkin lymphoma, prostate, and thyroid; localized: cervix, colon and rectum, endometrium, kidney/renal pelvis, non-Hodgkin lymphoma, oral cavity/pharynx, ovary, small intestine, and soft tissues

### Individual sustainability

Telephone surveys were pursued among the 100 participants who received the full 1-year intervention and completed either of the randomized controlled trials [19, 20]. Participants were asked, (1) “Have you continued to garden in the 12 months since the intervention has ended?” (yes or no) and, (2) “Have you expanded your vegetable garden space at all beyond what was initially set up as a part of the study?” (yes or no).

In addition, ACES maintains records of all Master Gardeners in the state. Thus, any participants who, after participation in *Harvest for Health*, went on to pursue Master Gardener training were documented.

### Organizational sustainability

Twenty-three stakeholders who were familiar with the *Harvest for Health* interventions were contacted via email in April of 2017 to complete an anonymous survey regarding the potential for organizational sustainability of the program. Stakeholders played instrumental roles in program planning, participant recruitment, intervention delivery, and their ongoing assistance in program evaluation. A representative sample of these stakeholders, comprised of ACES county agents and Master Gardener Program Coordinators (*n*=14), leaders of patient support groups (*n*=5), and UAB Cancer Care Network leaders (*n*=4), were among those contacted for the survey.

Organizational sustainability was assessed using the Program Sustainability Assessment Tool (PSAT). The PSAT is an instrument that was designed to be used by researchers, evaluators, program managers, and public health program staff to assess program capacity for sustainability [21, 22]. Program sustainability capacity is defined as “the ability to maintain programming and its benefits over time” [22]. The PSAT includes 40 items across eight domains (each with five items) and is based on a program sustainability framework [15]. The domains include environmental support, funding stability, partnerships, organizational capacity, program evaluation, program adaptation, communications, and strategic planning. Definitions of each domain are included in Table 2. All items are on a seven-point Likert scale (1 = “to little or no extent”; 7 = “to a great extent”). This tool provides an automated summary report of the program’s overall sustainability that can be used to engage in sustainability planning [23].

**Table 2:** PSAT sustainability domains and definitions (Center for Public Health Systems Science, 2013)

Domain	Definition	*Harvest for Health* PSAT Score (% of maximum score)
Environmental Support	Supportive internal and external climate for program, such as support or resources from champions or leadership within or outside of the organization; public support.	5.9 (84.2%)
Funding Stability	Establishing consistent financial base for program, such as variety, stability, and flexibility of funding sources.	5.0 (71.4%)
Partnerships	Cultivating connections between program and stakeholders, such as communication with or involvement with community leaders or commitment of community members.	5.2 (74.2%)
Organizational Capacity	Presence of internal support and resources necessary to effectively manage program, such as efficient management or staff and resources, adequate staff to complete program activities or goals.	5.8 (82.8%)
Program Evaluation	Assessing the program to inform planning and document results, such as quality program evaluation, reporting of short-term and intermediate outcomes, results shared with stakeholders, e.g., the public, funders, etc.	6.3 (90%)
Program Adaptation	Taking actions that adapt the program to ensure its ongoing effectiveness, such as adapting strategies if and when needed	6.2 (88.5%)
Communications	Strategic communication with stakeholders and the public regarding the program, such as increasing community awareness and generating interest, demonstrating the value of the program.	5.6 (80%)
Strategic Planning	Using processes that guide your program’s directions, goals, and strategies, such as plans for future resource needs, a sustainability plan, a long-term financial plan.	5.2 (74.2%)
**Overall Capacity**	**Total Overall Score**	**5.7 (81.4%)**

The PSAT has been developed and tested on a large number of programs at differing levels and has established reliability (Cronbach’s α ranging from 0.79 to 0.92 for each subscale; overall average of 0.88) [21]. Preliminary validation analyses suggest PSAT scores are related to important program and organizational characteristics [21].

### Statistical analyses

Descriptive statistics that relied on the generation of frequencies, percentages, and means were used to analyze the data for both individual sustainability and organizational sustainability. Univariate logistic regression was used to identify individual predictors of continued gardening and garden expansion, which included sociodemographic and health-related characteristics collected prior to intervention delivery (data collected previously described [18]). For each PSAT domain, the average score among the completed items was calculated. The overall capacity was calculated as the average of the 8 domains scores. Data were analyzed using SPSS (version 24).

## Results

### Sustainability indicators of individual participant behavior

Of the 100 cancer survivors who were identified for the individual sustainability survey, one was deceased, and 8 were unable to be contacted (91.9% response rate). Respondents of the survey were primarily female and breast cancer survivors (96.7% and 93.4%, respectively per study design), non-Hispanic White (76.9%), and resided in urban counties (78.0%). The mean age was 63.0 years (SD 9.6). On average, the sample reported 4.5 (SD=2.6) functional limitations and 3.6 (SD=2.6) comorbidities at the start of the feasibility trials.

Of the respondents, 78 (85.7%) continued gardening throughout the 12 months following the completion of the intervention. Further time since diagnosis and receipt of chemotherapy were the only sociodemographic or health-related characteristics associated with reduced likelihood of continued gardening (OR=0.87; 95% CI=0.78-0.98 and OR=0.12 95% CI=0.02-0.99, respectively). Of those who continued gardening, 43 (47.3%) had expanded their gardens beyond the initial 4x8 foot raised bed or 4 Earthboxes® (Novelty Manufacturing Co., Lancaster, PA) provided by the project. Participants who had received chemotherapy were less likely to report having expanded their garden (OR=0.31; 95% CI=0.13-0.76). For those who did not elect to expand their gardens, roughly half stated that they planned to do so in the future, and the other half were satisfied with maintaining the current space they had (one participant reported that while they did not expand their space, they helped their daughter establish a garden in her yard). Among the 13 participants who reported discontinued vegetable gardening, reasons for discontinuation included illness of self or partner (*n* = 5), plans to return to gardening in the future (*n* = 2), preference for flower gardening (*n* = 1), moving homes (*n* = 1), lack of time (*n* = 1), lack of interest (*n* = 1), discouragement after bad gardening seasons (*n* = 1), and unknown (*n* = 1).

Five participants had begun the process or had already become Master Gardeners post-intervention. In Alabama, this process requires completion of fifty hours of instruction in gardening practices and pest control followed by fifty hours of volunteer community service utilizing the training. This is followed by the expectation that Master Gardeners volunteer at least fifty hours annually to maintain certification. This interest and time investment is a testament to not only the ability of the *Harvest for Health* intervention to encourage a lasting behavior change but also to the skills developed during the intervention with mentoring by the Master Gardeners.

### Indicators of organizational sustainability

Twenty-one of the 23 program stakeholders completed the PSAT (91.3% response rate). Eleven stakeholders were ACES county agents who, as a part of this program, assisted in recruitment, screening, training, and supporting Master Gardener volunteers who agreed to become mentors for the project. Two stakeholders were Master Gardener Program Coordinators who aided in communications between all parties of ACES and made local visits to support mentor recruitment and training. Five stakeholders were leaders of patient support groups that *Harvest for Health* had worked with for recruitment and education purposes: FORGE (a breast cancer support group in the metropolitan Birmingham area) (*n* = 2); Susan G. Komen Breast Cancer Foundation (*n* = 1); UAB Cancer Center Education & Support Services (*n* = 1); and the St. Vincent’s Bruno Cancer Center Support Group (*n* = 1). Three stakeholders were members of the UAB Cancer Care Network, satellite hospitals throughout the southeast U.S. that were key in referring cancer survivors to *Harvest for Health*.

### PSAT scoring

*Harvest for Health* received a 5.7 out of 7 (81.4% of the maximum score) in overall capacity for sustainability. All PSAT scores and the domain descriptions are reported in Table 2. According to stakeholders, *Harvest for Health’s* strongest scores were in the domains of Program Evaluation, Program Adaptation, Environmental Support, Organizational Capacity, and Communication (scores ranging from 5.6 to 6.2, representing 80-89% of the maximum score). Its weakest scores, while still relatively high, were in the domains of Partnerships, Strategic Planning, and Funding Stability (scores ranging from 5.0 to 5.2, representing 71-74% of the maximum score).

## Discussion

To our knowledge, this is one of the first studies to demonstrate the capacity for sustainability of a behavioral intervention among cancer survivors and key stakeholders. Moreover, it is likely to be the very first to collect such data on a vegetable gardening intervention that focuses on this patient population.

The resulting data suggest that *Harvest for Health* is successful in engaging participants in the intervention behavior (i.e., gardening) that lasts long beyond the initial 12-month intervention. Based on recent reviews, few diet and physical activity interventions report post-intervention follow-up, and even fewer report long-term follow-up (≥12 months). For example, only 4 of 14 studies reported on maintenance of functional change after home or community-based physical activity interventions, with follow-up ranging from 12 to 26 weeks post-intervention [24]. The lack of interventions examining long-term lifestyle behavior change (12 months or longer) among cancer survivors has been identified as an important research gap [8, 24].

In contrast, the majority of those enrolled in *Harvest for Health* continued to garden one-year beyond intervention completion. Furthermore, we found that nearly half of participants drew from their own resources (e.g., time, money, space) to expand their gardens. This investment on the part of the prior participants speaks not only to their commitment to the vegetable gardening intervention but also to the skills they learned and retained from their Master Gardener mentors. Moreover, while the telephone surveys did not systematically ascertain reasons for continued gardening (as it did for discontinuation of gardening), it is likely that intervention participants experienced the benefits reported in these two studies. Benefits included increased reassurance of worth (i.e., feeling of adding value or deserving a place in society), improved physical performance and the production of fresh vegetables which improved their diets [19, 20].

One of the core strengths of *Harvest for Health* regarding sustainability is that Master Gardeners are volunteer workers who depend on annual hours of education and volunteer work to maintain their accreditation status. While there are many volunteer opportunities to select from, an ACES survey indicated that Master Gardeners are motivated by volunteer activities that allow them to help other people, and to learn and advance their knowledge and skills, especially through hands-on experiences. Importantly, with some participants becoming Master Gardeners, there will be more of a connection between the program and the community (Partnerships), more resources (Master Gardener mentors) to effectively manage the program and its activities (Organizational Capacity), and more people to strategically disseminate the program outcomes and activities to stakeholders, decision-makers, and the public (Communications).

In terms of organizational sustainability, *Harvest for Health* demonstrated several notable strengths. First, its overall capacity for sustainability was robust (5.7 out of 7.0 points), suggesting this program has strong capacity for sustainability, especially when compared against an average score of 4.84 rendered in a review of 252 various public health programs by Luke et al. [21]. It is noted that these 252 programs involved tobacco use, diabetes, obesity, oral health, and multiple health topics. Comparing *Harvest for Health* to more similar programs, namely health promotion interventions for cancer survivors, would likely be more appropriate; however, as mentioned, few other studies have been performed.

The specific strength of the program in the domain of Program Evaluation is substantiated given the regular meetings with funders and stakeholders in which program staff report outcomes, use evaluation results to inform planning and implementation, and demonstrate successes of the program. Also, outcomes of the program are regularly presented at national meetings and provided to the public in the form of media (e.g., on-air news, newspaper articles, online news) and social network (i.e., public Facebook page, ACES website). While qualitative outcomes related to satisfaction are important, quantitative outcomes are of primary interest to the Master Gardener Programs for evaluation. Given the Programs’ priority of teaching home gardeners to become successful growers, the key outcomes from Harvest for Health were sustainment of gardening and expansion of garden size by cancer survivors one year after the study ended. Thus, these activities and outcomes likely contributed to the strong score.

Program Adaptation, another strength, was likely the result of several factors. First, , *Harvest for Health* was designed from the start to be delivered by Extension Master Gardeners – a decision that was informed by early involvement of ACES (state agency and a local county agent). Thus, *Harvest for Health* likely required fewer and simpler adaptations compared to interventions designed to assess efficacy that consider sustainability late in the program’s life cycle. Second, the minor adaptations that have been made to the program were made through an iterative process to further improve the fit to the intended recipients, both cancer survivors as well as the Master Gardener Program. Any components of the program that have been found ineffective or too expensive (price increases) are regularly assessed and de-implemented or replaced (i.e., removal of soaker hoses for those with Earthboxes®, provision of garden tools, e.g., spades and garden hoses, only to those who did not already have them) in order to reduce costs. Conversely, deer fences and frost cloths were found to be necessities for some survivor gardens and were added.

Environmental Support emanated from the champions who strongly supported the program, who were likely to be individual Master Gardeners, but also the leadership support within the larger organization of ACES. Master Gardeners, many with family and friends interested in *Harvest for Health*, wanted to help publicize the study or recruit additional participants. They also provided extra support through donating extra seeds on hand, extra transplants they grew, or in some cases, retail purchases for an extra item they deemed important for their participant’s success in gardening. In addition, support was also provided by the O’Neal Comprehensive Cancer Center at UAB, other cancer support groups, as well as strong public support (interest generated via word of mouth). Donations or discounts on gardening supplies for the study were provided by local retailers such as independent nurseries and hardware stores, and individual Walmart® stores ($50 donations to support the purchase of gardening gloves and seeds), charities, and other interest groups. Master Gardeners who enjoy working with the program regularly approach *Harvest for Health* for future volunteer opportunities. Moreover, previous participants, i.e., cancer survivors, have called in to see if they can participate again.

Organizational Capacity is evident in the strong support and resources within the ACES Master Gardener Program to effectively deliver *Harvest for Health*. Organizational systems are in place to support the various program needs, leadership effectively articulates the vision of the program to external partners (e.g., recruitment events), leadership efficiently manages staff and other resources (no staff are hired without guaranteed pay; donations are sought for extra study items – i.e., sunscreen and hats), and *Harvest for Health* has adequate staff to complete the goals of the program (ACES, Master Gardeners).

Regarding Communications, there were many presentations at local and state Master Gardener meetings and conferences, as well as cancer-related venues that were convened at chapters of community-based support groups, public health offices, and hospitals. While initially there had been less communication with the general public, strides have already been made to secure and maintain public support. Notably, a publicly available Facebook page, https://www.facebook.com/harvesting4health/ is being monitored by ACES and *Harvest for Health* staff to allow anyone to share his or her gardening experience to the benefit of others. Improvements can be made in communicating the need for the program to the public (establishing more of a media presence) and marketing in a way that generates interest (targeting our population with messages of interest). Additionally, strategies are needed to increase community awareness of the issue (educating cancer survivors about increased risk of second cancers and other diseases and functional decline), and demonstrating the program’s value to the public (more coverage of previous participants’ benefits gained).

Although PSAT scores were strong overall, they also speak to areas where the program can be improved. The weaker scoring domains of the PSAT were Strategic Planning, Partnerships, and Funding Stability. These will be areas for improvement and growth. For Harvest for Health, the greatest investment in time for the Master Gardener Program has been recruitment of volunteers and monitoring fidelity via monthly check-ins. Strategic Planning after the feasibility studies has led to processes to address these and other activities. For example, one central person was hired to manage the logistics and communication. Also, there has been increased and repeated promotion of the study at Master Gardener Program trainings and meetings. Areas for improvement include better planning and communication of future needs for resources and a long-term financial and sustainability plan. Furthermore, making sure that the program’s goals are well understood by all stakeholders and clearly outlining the roles and responsibilities of all stakeholders will improve the program and increase capacity for sustainability.

*Harvest for Health* is involved with many community organizations currently, and has established strong Partnerships with FORGE (a local breast cancer support agency) and the Jefferson County Master Gardener Program in which the intervention is disseminated more broadly. However, the intervention’s Partnerships could be further strengthened through forging connections with more diverse community organizations. For example, more community organizations are needed that can provide garden supplies, plants, and seeds. While the garden boxes worked well for many participants with limited space, not all apartment facilities are able to accommodate these small containers. Therefore, organizations that could offer space for garden plots to these individuals would help to bring this intervention to scale. Community leaders play an important role in *Harvest for Health.* By better communicating with these leaders and getting them more involved in the program in a more visible way, community members will be more committed to and engaged in the program and its goals.

Funding stability is a concern for many programs like *Harvest for Health*. Locally, a community organization, the Women's Breast Health Fund of the Community Foundation of Greater Birmingham, has provided scholarships to encourage breast cancer survivors who cannot afford gardening supplies and wish to reap the benefits evidenced by this intervention. In addition, ACES reduced the cost of Master Gardener credentialling by 25% and fundraising efforts through UAB have resulted in further scholarships to cancer survivors who have completed the program. As *Harvest for Health* initially began as a small pilot study, it is understandable that stakeholders may have considered the program transient; however, it has grown over time to a statewide initiative, and is currently being evaluated in the Southwest [25, 26]. To facilitate further dissemination to other regions of the country, with the goal of eventually expanding nationwide, a long-term stable funding mechanism will need to be put in place.

While this study is one of the first to document individual and organizational sustainability and does so in a systematic manner with strong response rates, there are limitations. First, both surveys relied on self-reported data, and the survey conducted among stakeholders had a fairly small sample. Also, there is potential for demand effects as stakeholders may have provided a more positive evaluation of the program. Although stakeholder responses were aggregated and anonymous, with such a small sample, there is nevertheless the concern that they may not have felt completely free to evaluate the program openly. While thoughtful planning for sustainment early in the translation process is crucial for achieving public health impact, this requires an iterative process [27, 28]. This evaluation occurred early in the life cycle of *Harvest for Health*, in response to the feasibility studies in which the research team helped to support the delivery of the intervention by the Master Gardener Programs. Future evaluations will be needed as the Master Gardener Programs transition towards supporting all aspects of intervention delivery.

Next steps will include developing an action plan and then taking action. The action plan will involve assembling a planning team, reviewing the program mission and purpose, reviewing PSAT results, determining program elements that need to be maintained, eliminated, or adapted, and prioritizing areas of sustainability capacity to address first. Taking action will involve implementing the action plan and reassessing sustainability capacity annually [29]. Focusing on *Harvest for Health’s* weaker elements of capacity for sustainability, this will mean closely examining longer-term funding options and ensuring a strategic plan is not only in place, but made known to all involved. Lastly, closer partnerships will need to be formed and maintained with regular communication.

## Conclusions

This paper examined *Harvest for Health’s capacity for* sustainability from the perspective of cancer survivors receiving the intervention (i.e., continued intervention behavior, transfer of skills) and the perspectives of various stakeholders. The majority of previous participants continued to engage in gardening and nearly half expanded their gardens so that they could engage on a larger scale, which means they are more likely to benefit long-term from the intervention’s health benefits. Additionally, some cancer survivors who completed the intervention went on to become Master Gardeners in an effort to continue the program. The general desire of previous participants to pursue skills and gain experience shows promise for multiple aspects of sustainability (e.g., organizational capacity, communications, strategic planning). From stakeholders’ perspectives, program evaluation was considered *Harvest for Health’s* strongest component, while funding stability was considered its weakest, though still favorable. In sum, *Harvest for Health* has many strengths in regard to capacity for individual-level and organizational sustainability. Future research will capitalize on the present research to maximize this novel intervention’s reach and impact on the steadily increasing population of cancer survivors in the United States.

## Data Availability

Both proposals that informed this project neither involved the development of model organisms nor were genome-wide association studies. Moreover, the budgets for each project fell far below the $500,000 cap where data sharing is required and available resources are provided for such purposes. The data that support the findings of this study are available from the University of Alabama at Birmingham (UAB) but restrictions apply to the availability of these data, which were used under license for the current study, and so are not publicly available. Deidentified data are however available from one of the authors (WDW) upon reasonable request and with permission of UAB.

## Abbreviations

ACES: Alabama Cooperative Extension System
Extension: Cooperative Extension System
PSAT: Program Sustainability Assessment Tool
UAB: University of Alabama at Birmingham
